# Different Roles of Introgression on the Demographic Change in Two Snakebark Maples, *Acer caudatifolium* and *A. morrisonense*, with Contrasted Postglacial Expansion Routes

**DOI:** 10.3390/plants11050644

**Published:** 2022-02-26

**Authors:** Min-Xin Luo, Yi-Ting Tseng, Jui-Tse Chang, Chien-Ti Chao, Pei-Chun Liao

**Affiliations:** Department of Life Science, National Taiwan Normal University, Taipei 116059, Taiwan; xji6aup3vup@gmail.com (M.-X.L.); subira0034@gmail.com (Y.-T.T.); b03612004@ntu.edu.tw (J.-T.C.); ff8bahamut@gmail.com (C.-T.C.)

**Keywords:** *Acer*, genetic admixture, glacial bottleneck, introgression, postglacial range expansion, secondary contact

## Abstract

Hybridization frequently occurs in plant species. With repeated backcross, the introgression may influence evolutionary trajectories through the entry of foreign genes. However, the genetic admixture via hybridization events is often confused with the ancestral polymorphism, especially in closely related species that have experienced similar evolutionary events. In Taiwan, two independent-originated endemic snakebark maples have contrasted postglacial range expansion routes: northward and upward expansion in *Acer caudatifolium* and downward expansion in *A. morrisonense*. The range expansion causes the current parapatric distribution, increasing the possibility of introgression. This study elucidates how their genetic variation reflects introgression and historical demography. With 17 EST-SSR markers among the intensely sampled 657 individuals, we confirmed that the genetic admixture between species mainly was attributed to recent introgression instead of common ancestral polymorphism. The secondary contact scenario inferred by approximate Bayesian computation suggested that *A. morrisonense* received more genetic variations from *A. caudatifolium*. Introgression occurred in colonized Taiwan around the early Last Glacial Period. Furthermore, the demography of *A. caudatifolium* was more severely affected by introgression than *A. morrisonense*, especially in the wavefront populations with high altitude range expansion, implying an altitude-related adaptive introgression. In contrast, *A. morrisonense* exhibited ubiquitous introgression independent of postglacial expansion, suggesting that introgression in *A. morrisonense* was neutral. In terms of different genetic consequences, introgression had different demographic impacts on species with different altitude expansion directions even under the same climate-change conditions within an island.

## 1. Introduction

Hybridization in vascular plants is common [1] but not universal [2]. Perennials and outbreeding with characters of stabilized hybridity, such as agamospermy and vegetative spread, characterize hybrid hotspots [2]. Via hybridization, genes are invasive from other species [3], which is likely to prevent fixation [4] and slow the evolution of reciprocal monophyly [5] but less likely to cause the fusion of species [6]. Introgression can occur via repeated hybridization and backcross or through the easily permeable genome regions of species boundary. The former describes the phenomenon of incorporating alleles from one species into another, while the latter may, but not necessarily, imply the selectivity of the porous genome [5]. Heterogeneous recombination rates across the genome could also result in the semipermeable nature of species boundary [7]. In other words, introgression refers to the common phenomenon of foreign genes entering the gene pool of a species, but its mechanism may be complicated.

When the environment changes, life will find a way to survive through changes in the genetic material (selection) or movement (migration or dispersal). The postglacial recolonization accelerated physical contact, promoting gene flow between lineages, namely “introgression”. For example, range expansion from different refugia caused genetic admixtures in European oaks [8,9] and maples [10]. Range expansion facilitates introgression by secondary contact [11], increasing the heterospecific density in sympatry, which reduces the pollen deposition rate [12] and promotes interspecific pollen transfer [13]. Interspecific pollen transfer is a mechanism of character displacement in habitat affinity and pollinator switches, which accelerates colonization and range expansion [13]. Introgression may also widen niches [14] and facilitate range expansion [15], especially when facing climate change [16]. In other words, range expansion accelerates introgression [17], increasing genetic variation and widening niches [14], resulting in positive feedback.

Under global climate change, species distribution ranges may shift. Compared with flat-continent organisms, the mountain species inhabiting different climatic zones are more likely to escape from climate adversity by elevational migration. During this elevational range shift, the initially allopatric species may undergo secondary contact and cause hybridization, further alternating the demography, genetic diversity, and colonization routes. Consequently, understanding the evolutionary history and spatial heterogeneity of these events underpinned the conservation strategy in their potential hybrid zones.

**Figure 1:**
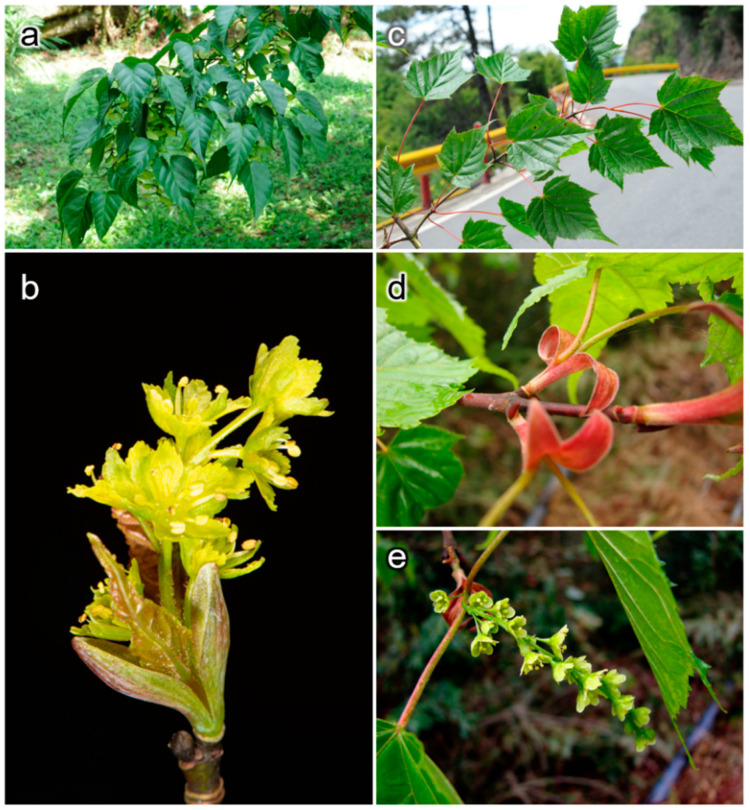
Leaf and floral morphology of *Acer caudatifolium* and *A. morrisonense*. (**a**,**b**) are the leaves and inflorescence of *A. caudatifolium*, respectively. (**c**–**e**) are the leaves, red stipules, and inflorescence of *A. morrisonense*, respectively. These two species have diagnostic characters of three-lobed vs. five-lobed leaves, yellowish green vs. red stipules, and short vs. long inflorescences between *A. caudatifolium* and *A. morrisonense*.

To study this issue, the two phylogenetic, closely related but paraphyletic maples *Acer morrisonense* and *A. caudatifolium* in Taiwan [18,19,20] were used. These two maples are endemic and least-concern (LC) species in Taiwan [21]. They have partially overlapped distributions and distinct morphological differences. *Acer morrisonense* grows in the forest from middle to high elevations, while *A. caudatifolium* has a broader altitudinal range from low to high elevations. *A. morrisonense* leaves are five-lobed and *A. caudatifolium* leaves are 3-lobed. Petals are oval with wavy margins in *A. morrisonense*, but rhomboid and serrate in *A. caudatifolium*. Inflorescence of *A. morrisonense* (ca. 6~10 cm) is longer than *A. caudatifolium* (ca. 5 cm) (Figure 1). Individuals with intermediate morphologies were found in the sympatric populations. 

**Figure 2:**
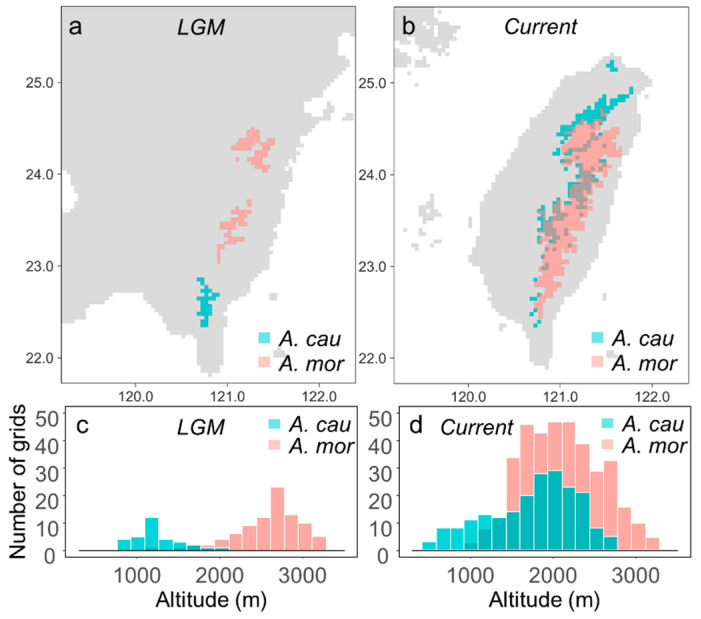
Geographic distributions of *Acer caudatifolium* and *A. morrisonense* inferred by ecological niche modeling (ENM). (**a**,**b**) are the most proper geographic distributions in the Last Glacial Maximum (LGM) and the present, respectively. (**c**,**d**) denote the altitudinal distribution in the LGM and the present, respectively. The grid numbers of (**c**,**d**) were counted from (**a**,**b**), respectively. These plots showed the allopatric distribution of two maples in the LGM and the current parapatric distributions. Data on ENM distributions are adopted from Luo et al. [22] and Chang et al. [23].

Besides, the two species dramatically changed in spatial and genetic distributions from the Last Glacial Maximum (LGM) to the present [22,23]. During the LGM, *A. morrisonense* was distributed at higher-elevational refugia, and *A. caudatifolium* was confined in the low mountains of southern Taiwan (Figure 2). Then, the postglacial warming relaxed the pressure of harsh environments on their survival, leading to rapid expansion. *Acer morrisonense* was downward shifting in range [23], while *A. caudatifolium* was upward- and northward-moving after the LGM [22]. The postglacial range shifts cause elevational overlap in the distribution of these two maples (Figure 2), leading to the probability of hybridization or introgression.

In this study, using genotyping data of these two maple species, we aimed to answer the following questions: (1) How are the degrees of genetic admixture between these two maple species? (2) Is the genetic admixture between these two maples from a common ancestral polymorphism or a result of recent genetic introgression? (3) If the introgression occurred, how does introgression influence the demographic dynamics in terms of effective population size change? Furthermore, (4) are these introgressions related to the postglacial range expansion of two maples in Taiwan? This study characterizes the multilocus diversity pattern of introgression to explore the demographic change after secondary contact.

## 2. Materials and Methods

### 2.1. Sampling and DNA Extraction

In total, we sampled 657 samples in Taiwan for SSR genotyping, in which 371 individuals of *A. caudatifolium* were collected from 20 populations, and 286 individuals of *A. morrisonense* were from 19 populations (Table S1). The genomic DNA was extracted from the fresh leaves using modified Cetyltrimethylammonium bromide (CTAB) approach [24,25]. Polyvinylpyrrolidone (PVP) and Polyvinylpolypyrrolidone (PVPP) were added to remove polysaccharides, phenolic compounds, and other secondary metabolites. RNaseA was used to remove excess RNA. NanoDrop microvolume spectrophotometer was used to qualify and quantify the extracted DNA.

### 2.2. Transferable Expressed Sequence Tag-Simple Sequence Repeat (EST-SSR)

The primers for the microsatellite DNA (simple sequence repeats, SSR) were designed from putative transcripts (Source Version: 01052015) of *A. saccharum* in Hardwood Genomics Project (https://www.hardwoodgenomics.org/) (accessed on 24 July 2017) using SciRoKo v3.4 [26]. We set the criteria of two-or-more nucleotide motifs with at least four repeats to select the SSR loci. All selected loci were compared with the transcriptome of *A. negundo* (Accession: VFFP) in One Thousand Plants (1KP) Consortium (https://sites.google.com/a/ualberta.ca/onekp/) (accessed on 30 August 2017), and the E-value must be <1 × 10^−10^. The lengths of all SSR loci were <500 bps with 40~60% GC content. The designed primers were checked with IDT Oligo Analyzer 3.1 (http://sg.idtdna.com/calc/analyzer) (accessed on 20 September 2017), and the differences of the annealing temperatures were <3 °C between forward and reverse primers. In total, we designed 47 primer pairs, of which 29 primer pairs were amplifiable. However, nine of 29 SSR loci were monomorphic between species, and three of the remaining generated > 70% null alleles. A total of 17 available SSR loci were obtained finally. All primer pairs were published in Chang et al. [23].

PCR products of these 17 loci were genotyped by capillary electrophoresis on an Applied Biosystems 3730 DNA Analyzer (Applied Biosystem, San Francisco, CA, USA). Fragment sizes of SSR alleles were read by Peak Scanner version 1.0 (Applied Biosystem, San Francisco, CA, USA) and corrected by reference to the size standard ABI GS500 LIZ (Applied Biosystems, San Francisco, CA, USA) with the assistance of the National Center for Genome Medicine, Academia Sinica, Taiwan. Peaks with a minimum height of 100 were read, and the sizes falling into the expected range were manually checked and adjusted. Genotypes of *A. morrisonense* were used for the demographic analyses and published in Chang et al. [23].

### 2.3. Genetic Diversity Estimation

The observed (*Ho*) and expected heterozygosity (*He*), Wright’s inbreeding coefficient (*F_IS_*) of the SSR loci of each species were calculated by GenAlEx 6.502 [27]. The private alleles of each species were calculated. Linear regression was conducted to test the geographic relationship (altitude, longitude, and latitude) with private allele frequency. 

### 2.4. Testing Introgression by STRUCTURE

The genetic structure between the two species was assessed using the Bayesian clustering analysis (BCA) by STRUCTURE v. 2.3.4 [28,29]. The grouping numbers *K* = 1–21 were set with ten replications. Each replication conducted a 10^6^-times Markov chain Monte Carlo simulation with 20% burn-in. The best *K* was evaluated by Δ*K* and CV error in Structure Harvester [30]. We defined the admixture coefficient (*Q*) > 0.05 as the introgression. 

### 2.5. Approximate Bayesian Computation (ABC)

**Figure 3:**
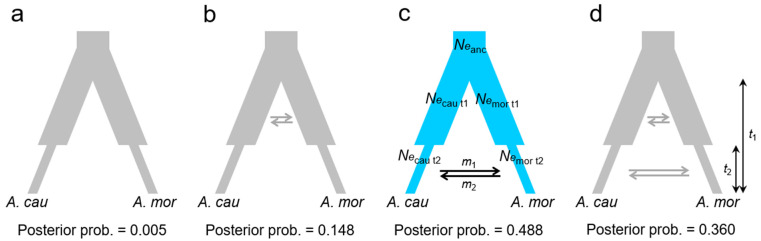
Four evolutionary scenarios describe all possible situations of interspecific gene flow after species divergence, including (**a**) no gene flow (**SI**), (**b**) early (**AM**), and (**c**) recent interspecific gene flow (**SC**), and (**d**) continuous gene flow after speciation (**CM**). The branch thickness represents the effective population size (*N_e_*) in the recent (*t*_2_), early (*t*_1_), and of the most recent common ancestor (i.e., the uppermost part where the branches converge). Horizontal arrows indicate the direction of gene flows from *Acer caudatifolium* to *A. morrisonense* (*m*_1_) and the opposite direction (*m*_2_). Model parameters *N_e_* and *m* denote the effective population size and migration rate. In these scenarios, two species diverged at *t*_1_ ago, and their population sizes were allowed to change at *t*_2_, the time of stopping interspecific gene flow in (**AM**) scenario or starting to gene flow in the (**SC**) scenario. Abbreviations of four scenarios: (**SI**): strict isolation, no interspecific gene flow was allowed after species divergence; (**AM**): ancient migration, interspecific gene flow was allowed at the early stage of species divergence; (**SC**): secondary contact, interspecific gene flow was allowed at recent periods; (**CM**): continuous migration, interspecific gene flow was allowed at all times after species divergence.

**Figure 4:**
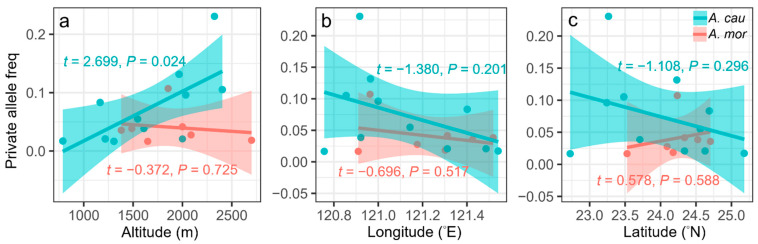
Geographic relationship with the private allele frequency of populations. The private alleles were predicted by (**a**) altitude, (**b**) longitude, and (**c**) latitude using linear regression.

Four evolutionary scenarios addressing the species divergence and interspecific introgression between parapatric species referred to [31] were tested by Approximate Bayesian Computation (ABC) using ABCtoolbox [32]: (1) strict isolation (SI) model: no gene flow after speciation (Figure 3a); (2) ancient migration (AM) model: gene flow occurred during initial divergence (Figure 3b); (3) secondary contact (SC) model: no gene flow during initial divergence but gene flow occurred in late periods of divergence (Figure 3c); (4) continuous migration after divergence (CM) model: gene flow persisted continuously during divergence (Figure 3d). These four scenarios described all possible situations in which two lineages coalesced back into a common ancestor and are mutually exclusive. The parameter setting of models is shown in Table S2. We performed 10^6^ simulations in fastsimcoal2 [33], and the best 2000 simulations were retained using the R package abc [34] to perform model selection with the neuralnet method. The model with the highest posterior probability (PP) and Bayes factors was suggested as the optimal scenario. Goodness-of-fit test was also used to confirm the preferred model for the observed data. Parameters of effective population size (*Ne*_anc_, *Ne*_cau_, *Ne*_mor_: effective population sizes of the common ancestor, *A. caudatifolium*, and *A. morrisonense*, respectively), migration rate (*m*_1_, *m*_2_), and divergence time (*t*_1_), and the time to gene flow (*t*_2_) of the optimal model were calculated. On the basis of these four scenarios, we allowed a changing effective population size (*N_e_*) after species divergence at *t*_2_. 

## 3. Results

### 3.1. Genetic Diversity

Each of *A. caudatifolium* and *A. morrisonense* have one intraspecific monomorphic locus among the 17 interspecific polymorphic SSR loci. There is no significant difference in the genetic diversity between these two species estimated by SSR: the *Ho* and *He* are 0.169 ± 0.044 and 0.341 ± 0.073 in *A. caudatifolium* and 0.180 ± 0.017 and 0.261 ± 0.062 in *A. morrisonense*, respectively (Table 1). However, relatively abundant private alleles were detected in *A. caudatifolium* than *A. morrisonense* (Table 1). A further linear regression showed a positive association of private allele frequency of populations with altitude in *A. caudatifolium* (*t* = 2.699, *p* = 0.024, Figure 4a), but not in *A. morrisonense* (*t* = −0.372, *p* = 0.725, Figure 4a). Neither longitude nor latitude was related to the private alleles (Figure 4b,c). Detailed estimates of genetic diversity in each population are listed in Table S3. The pairwise *F*_ST_ among populations between the two species ranges 0.249–0.718. The average pairwise *F*_ST_ among populations of *A. caudatifolium* (*F*_ST_ = 0.144) is higher than *A. morrisonense* (*F*_ST_ = 0.106, *p* = 1.5 × 10^−11^, Table S4). 

Analysis of molecular variance showed a significant divergence between two species (*Φ*_CT_ = 0.478, *p* < 10^−5^) and among populations (*Φ*_SC_ = 0.149, *p* < 10^−5^) (Table 2). In terms of individual species, most genetic variation existed within populations (82.5% and 89.9% in *A. caudatifolium* and *A. morrisonense*, respectively), but there was still significant genetic differentiation between populations (*Φ*_ST_ = 0.175 and 0.101 in *A. caudatifolium* and *A. morrisonense*, respectively, Table 2). The significant population differentiation reflected the positive inbreeding coefficient estimates (*F*) in both species. 

### 3.2. Genetic Introgression

The BCA showed an apparent genetic grouping between the two maples. However, some individuals still had a few genetic components of another species. Five individuals of *A. morrisonense* were suggested to be misidentifying in morphology because of the high genetic component of *A. caudatifolium* (92.29~99.80%), and one sample with 65.74% component of *A. caudatifolium* was assigned as a hybrid individual. Taking *Q* > 0.05 as the threshold, 9 of the 371 *A. caudatifolium* (2.43%) and 15 of the 281 *A. morrisonense* (5.34%) were genetically introgressive. Notably, these introgression events were not confined to specific populations but were occurred in sympatric (altitudinal parapatric) populations. This analysis implied that introgression was recent, common, and seemed asymmetric from *A. caudatifolium* toward *A. morrisonense* in Taiwan.

**Figure 5:**
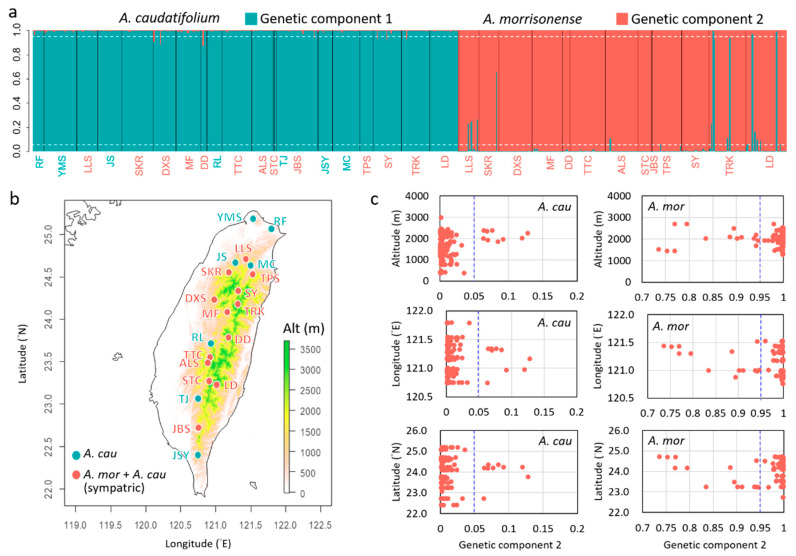
Genetic admixture patterns between *Acer caudatifolium* and *A. morrisonense*. (**a**) The Bayesian clustering analysis (STRUCTURE) shows the genetic admixture between species. The dotted line indicated the introgression threshold of 5% genetic components of the other species. Populations abbreviated in red indicate sympatric distribution in two species. (**b**) Geographic distribution of sampling sites. Populations denoted in red are the sympatric populations. (**c**) Altitudinal, longitudinal, and latitudinal distribution of the genetic component 2 (the red component in (**a**)) in two maple species. The left panels are distributions of *A. caudatifolium,* and the right panels are distributions of *A. morrisonense*. The blue dotted line indicates the introgression threshold of 5% genetic components of the other species. The misidentified samples of *A. morrisonense*, i.e., >50% genetic components of *A. caudatifolium*, are not shown in (**c**).

When mapping the genetic components along longitude and latitude, we found that introgression in both species spanned a wide range of longitudes (120.74~121.34° E in *A. caudatifolium*; 120.87~121.52° E in *A. morrisonense*) and latitudes (22.72~24.35° N in *A. caudatifolium*; 23.23~24.72° N in *A. morrisonense*) in Taiwan. Introgressions also occurred across wide-range elevation (1450~2700 m above sea level, a.s.l.) in *A. morrisonense* but were restricted to the only higher elevation (1854~2375 m a.s.l.) in *A. caudatifolium* (Figure 5c), implying selectively (or adaptively) in *A. caudatifolium*.

### 3.3. Approximate Bayesian Computation

The genetic admixture that occurred early or recently after species divergence was tested in four evolutionary scenarios of isolation-with-migration by the approximate Bayesian computation (ABC) (Figure 3). Among the four scenarios, the strict isolation (SI) that did not allow any gene flow between species achieved the lowest posterior probability (PP = 0.005), while the secondary contact (SC) allowing interspecies gene flow at the late stage of divergence achieved the highest PP (PP = 0.488). The continuous migration (CM, PP = 0.360) and ancient migration (AM, PP = 0.148) scenarios achieved the second and third high PP, respectively. The extremely smaller PP in the SI scenario from the other three scenarios with gene flow showed that the gene flow continued after speciation, i.e., porous genomes between these two maples. The SC scenario also provides a good fit to the observed data (*p* = 0.45, Figure S1). Pairwise model comparisons between scenarios by Bayes factor were listed in Table S5. 

Under the best scenario SC, the interspecific gene flow was small but significant from *A. caudatifolium* to *A. morrisonense* (*m*_1_ = 8.0 × 10^−5^, 95% confidence interval (95%CI) = 5.0 × 10^−5^~3.3 × 10^−4^, Table 3, Figure S2), but the opposite directional gene flow was relatively small and cannot deviate from 0 (*m*_2_ = 2.0 × 10^−5^, 95%CI = 0~5.7 × 10^−4^, Table 3, Figure S2), in consistency with BCA results. Such an asymmetric gene flow occurred since 18,870 generations ago (95%CI = 14,067~66,799, Table 3, Figure S2). Comparing to the long-term divergence (18.74 Mya) estimated from cpDNA and nuclear ITS sequences [19], a relatively recent divergence time (*t*_1_ = 22,925 generations ago, 95%CI = 9349~27,664, Table 3, Figure S2) was estimated when using the coalescence approach by SSR marker. The current effective population sizes of *A. caudatifolium* (*Ne*_cau t2_) and *A. morrisonense* (*Ne*_mor t2_) were 1792 (95%CI = 1441~1854) and 366 (95%CI = 256~381), respectively (Table 3, Figure S2). The earlier *Ne* was 4.2-fold and 58.0-fold larger than the current *Ne* of *A. caudatifolium* (*Ne*_cau t1_ = 8232, 95%CI = 2848~9750) and *A. morrisonense* (*Ne*_mor t1_ = 24,220, 95%CI = 2495~38,419) (Table 3). The effective population size of the common ancestor was 408.7-fold large (*Ne*_anc_ = 149,670, 95%CI = 39,472~187,873, Table 3), revealing the long-term coalescence process between two lineages with a non-sister relationship. 

**Figure 6:**
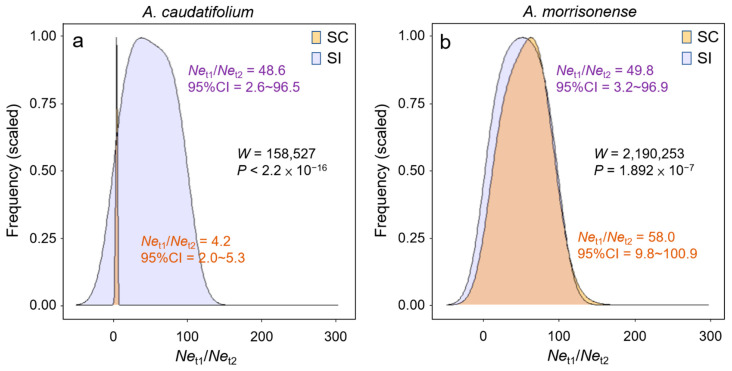
Scaled density plots of the estimated ratio of population size change (*Ne*_t1_/*Ne*_t2_) in (**a**) *Acer* *caudatifolium* and (**b**) *A. morrisonense* under the secondary-contact (SC) and strict-isolation (SI) scenarios. The significance of differences between simulations of different scenarios was conducted by Mann–Whitney U Test.

To confirm whether the population decline was introgression-related, we compared the amplitude of population decline (i.e., the ratio *Ne*_t1_/*Ne*_t2_) between scenarios with and without interspecific gene flow (i.e., SC vs. SI) (Figure 6). In *A. morrisonense*, the amplitude of population decline was severer in SC (*Ne*_t1_/*Ne*_t2_ = 58.0, 95%CI = 9.8~100.9) than in the SI scenario (*Ne*_t1_/*Ne*_t2_ = 49.8, 95%CI = 3.2~96.9, *p* = 1.892 × 10^−7^ in Mann–Whitney U test). In contrast, populations declined more severely in the SI (*Ne*_t1_/*Ne*_t2_ = 48.6, 95%CI = 2.6~96.5) than in the SC scenario (*Ne*_t1_/*Ne*_t2_ = 4.2, 95%CI = 2.0~5.3, *p* < 2.2 × 10^−16^). These estimates revealed that, after introgression, the population decline eased in *A. caudatifolium* but worsened in *A. morrisonense*.

## 4. Discussion

### 4.1. Secondary Contact during Colonization to Taiwan in Last Glacial Period

Past genetic hybridization studies on *Acer* section *Macrantha* mainly focused on phylogenetics and hybrid ancestry [18]. This study, via the best ABC scenario “Secondary Contact” and introgression components inferred by BCA, evidenced that genetic admixture comes from recent and ongoing introgression between *A. caudatifolium* and *A. morrisonense* rather than the ancestral polymorphisms. This continued introgression is not surprising, as *A. caudatifolium* and *A. morrisonense* bloom synchronously from March to April within a confined space of an island. Since these two island maples were allopatrically distributed during the LGM until the middle-Holocene range overlapping [22,23], we speculate that the recent introgression is due to the secondary contact since postglacial expansion. 

However, the onset time of introgression (*t*_2_) was longer than the end of LGM. In South Asia, the sea level rose at the rate of 0.41 m/100 yrs between 19.0 kya to 14.6 kya and accelerated to 5.33 m/100 yrs between 14.6 and 14.3 kya [35], suggesting the end of LGM at approximately 14~19 kya in Asia. Under the SC scenario, *t*_2_ was 18,870 generations ago, which must be earlier than the LGM unless there was one year per generation. However, although snakebark maples grow faster, according to Ďurkovič [36], *A. caudatifolium* takes at least five years to mature and flower. In other words, *A. caudatifolium* and *A. morrisonense* started introgression much earlier (*c.* 94 kya at the beginning of the Late Pleistocene) than the end of LGM if considering five years per generation.

The onset time of introgression roughly matches the beginning of the Last Glacial Period (LGP) of the Quaternary (*c.* since 110 kya), during which the sea level fell *>* 50 m lower than the present day [37], resulting in the exposure of the seabed (average depth < 50 m at present) of the Taiwan Strait (i.e., the Fujian–Taiwan land bridge or, namely, Dōngshān land bridge). Although unsure of the exact time, the land bridge formation benefited the island colonization of two maple species. We speculate that the interspecific gene flow is related to entering Taiwan. In other words, this secondary contact inferred by ABC does not refer to the post-LGM range expansion, but to the events of colonization to Taiwan.

### 4.2. Rapid Range Expansion Rather Than Hybridization Shaped the Genetic Diversity of Wavefront Populations

The founder effect of colonizing Taiwan also explains the severe population decline from *t*_1_ to *t*_2_ inferred by ABC. Although previous studies only backtracked paleo-distributions in LGM [22,23], these two species might also be confined within refugia since the earlier stage of the Quaternary LGP because of the continuous cold and aridity. However, we detected high frequencies of private alleles (Table 1), implying a rapid recovery of population size. We speculated that the reduced genetic diversity through population decline was supplemented by introgression, where the abundant private alleles reflect the rare-allele phenomenon after introgression [38]. If this hypothesis is supported, the population decline will be mitigated, *viz*., smaller *Ne*_t1_/*Ne*_t2_ in the SC scenario (allowing introgression) than in the SI scenario (not allowing interbreeding). 

However, the private allele frequencies were not high among hybrid populations (Table S3). In addition, the mitigation of population decline was only detected in *A. caudatifolium* but not in *A. morrisonense*. Unlike the prevalent introgression in *A. morrisonense*, *A. caudatifolium,* with lower introgression rates, received foreign alleles only at higher altitudes. If introgression can complement the reduced genetic diversity, the relaxation of demographic constraint should be more pronounced in *A. morrisonense*. Therefore, the hypothesis of genetic rescue by introgression could be rejected, and the abundant private alleles were alternatively explained by the rapid range expansion after LGM. Especially for *A. caudatifolium*, the positive association of private allele with altitude reflects its post-LGM upward expansion [22] because of the higher mutation rate in the wavefront of expanding populations [39].

Nevertheless, the population decline of *A. caudatifolium* under the SC model was indeed lessened than that without introgression. We also found more genetic admixture at high elevation, the wavefront of population expansion. Spatial expansion surfs not only rare alleles into newly colonized populations but also accelerates native genes into the invading species [17]. In other words, introgression may advance the upward expansion by supplying new genetic variation in *A. caudatifolium*. High proportional new alleles at high elevation may also reflect the altitude-related adaptive introgression in *A. caudatifolium* (Figure 4a). The introgression and increasing rare alleles are conducive to widening niches of frontier populations. Such a phenomenon was not rare. For example, alpine *Carex curvula* adapts to edge environments in marginal habitats by introgressive hybridization [14]; the Mexican teosinte introgressed from maize expanded its niches to facilitate the invasion in Europe [40]. Introgression improves the fitness of postglacial expanding species within the space-constraint island.

However, an abundance of rare alleles and introgressions in wavefront populations was not observed in *A. morrisonense*. In the ABC simulations, its population size declined more severely in the SC scenario than in the SI scenario, suggesting that introgression did not rescue the irreparable genetic loss of critical range shrinkage in the middle Holocene [23]. High proportional private alleles were not detected in wavefront and introgression populations and were related to neither longitude, latitude, nor altitude. Thus, despite being more receptive to foreign genes than *A. caudatifolium*, it is not reflected in adaptiveness as with *A. caudatifolium*. Chang et al. [23] also suggested that adaptation-independent pollen flow homogenized genetic differences between distant populations whose seed colonization was restricted by mountain barriers. This also indicates that *A. morrisonense* is more receptive to pollens of foreign genotypes, implying a higher chance of neutral introgression. 

## 5. Conclusions

In conclusion, this study proves that the genetic admixture of *A. caudatifolium* and *A. morrisonense* was attributed to the recent introgression of colonizing Taiwan since the LGP instead of from common ancestral polymorphism. However, the introgression did not recover the genetic loss of the LGP bottleneck effect. The increasing private alleles can only explain the post-LGM range expansion but not the rare allele phenomenon of introgression. However, the high-elevational introgression may adaptively facilitate upward range expansion of *A. caudatifolium*, but such a phenomenon was not found in *A. morrisonense* whose introgression seems to be neutral and unrelated to its postglacial range expansion. This study outlined the spatiotemporal genetic changes of the introgression between these two island endemic maple trees following entering Taiwan and presented arguments for their different adaptive propensities during introgression. 

## Figures and Tables

**Table 1:** Summary results of the estimated genetic diversity of *Acer caudatifolium* and *A. morrisonense*.

	*A. caudatifolium*	*A. morrisonense*
Parameters	Mean	Mean
Sample size	371	286
Number of alleles	146	106
Private alleles	61	21
*Ho*	0.169 ± 0.044	0.180 ± 0.050
*He*	0.341 ± 0.073	0.261 ± 0.062
*F*	0.366 ± 0.082	0.398 ± 0.088

**Table 2:** Analysis of molecular variance (AMOVA) between two maple species.

Source of Variation	df	SS	Var Comp	Var%	*Φ*
*A. caudatifolium* and *A. morrisonense*					
Among spp.	1	1614.35	2.464	47.789	0.478 *
Among pop within spp.	32	557.22	0.400	7.764	0.149 *
Within pop	1280	2935.94	2.292	44.447	0.556 *
Total	1313	5107.51	5.157		
*A. caudstifolium*					
Among pop	19	412.72	0.526	17.488	0.175 *
Within pop	351	1790.28	2.480	82.512	
Total	370	2203.00	3.005		
*A. morrisonense*					
Among pop	12	144.50	0.231	10.137	0.101 *
Within pop	273	1145.66	2.049	89.863	
Total	285	1290.16	2.281		

* *p* < 10^−5^, 9999 permutations.

**Table 3:** Model parameters of the SC scenario through approximate Bayesian computation. Details model parameters of four scenarios are listed in Table S6.

Scenario	*Ne*_anc_	*Ne*_cau t1_	*Ne*_mor t1_	*Ne*_cau t2_	*Ne*_mor t2_	*m*_1_	*m*_2_	*t*_1_	*t*_2_
mode	149,670	8232	24,220	1792	366	8.0 × 10^−5^	2.0 × 10^−5^	22,925	18,870
Wt. 2.5% perc.	39,472	2848	2495	1441	256	5.0 × 10^−5^	0	9349	14,067
Wt. 97.5% perc.	187,873	9750	38,419	1854	381	3.3 × 10^−4^	5.7 × 10^−4^	27,664	66,799

## Data Availability

The EST-SSR genotypes of *Acer caudatifolium* and *A. morrisonense* are available on Mendeley Data with http://dx.doi.org/10.17632/htpcv69w9z.1 (accessed on 1 February 2022) and Dryad with https://doi.org/10.5061/dryad.ghx3ffbng (accessed on 1 February 2022), respectively.

## Supplementary Materials

The following supporting information can be downloaded at: https://www.mdpi.com/article/10.3390/plants11050644/s1, Figure S1: Histogram of the distribution under secondary contact (SC) scenario and the observed value; Figure S2: The frequency of marginal density of the best-fit scenario “secondary contact” (SC); Table S1: Sampling sites and sample size; Table S2: Search ranges of parameters in ABC model; Table S3: Genetic diversity of each sampled population and species; Table S4: Pairwise *F*_ST_ between populations of two maple species; Table S5: Model comparison between four scenarios with the Bayes factor; Table S6: Estimates of model parameters for the four scenarios through approximate Bayesian computation.
