# Supplementary material for: Different Roles of Introgression on the Demographic Change in Two Snakebark Maples, Acer caudatifolium and A. morrisonense, with Contrasted Postglacial Expansion Routes
Source: Plants (Basel). 2022 Feb 26;11(5):644. doi: 10.3390/plants11050644 (PMC8912722; doi:10.3390/plants11050644)
Supplement: Supplementary file 1 [file plants-11-00644-s001.zip › plants-1607902-supplementary.pdf]

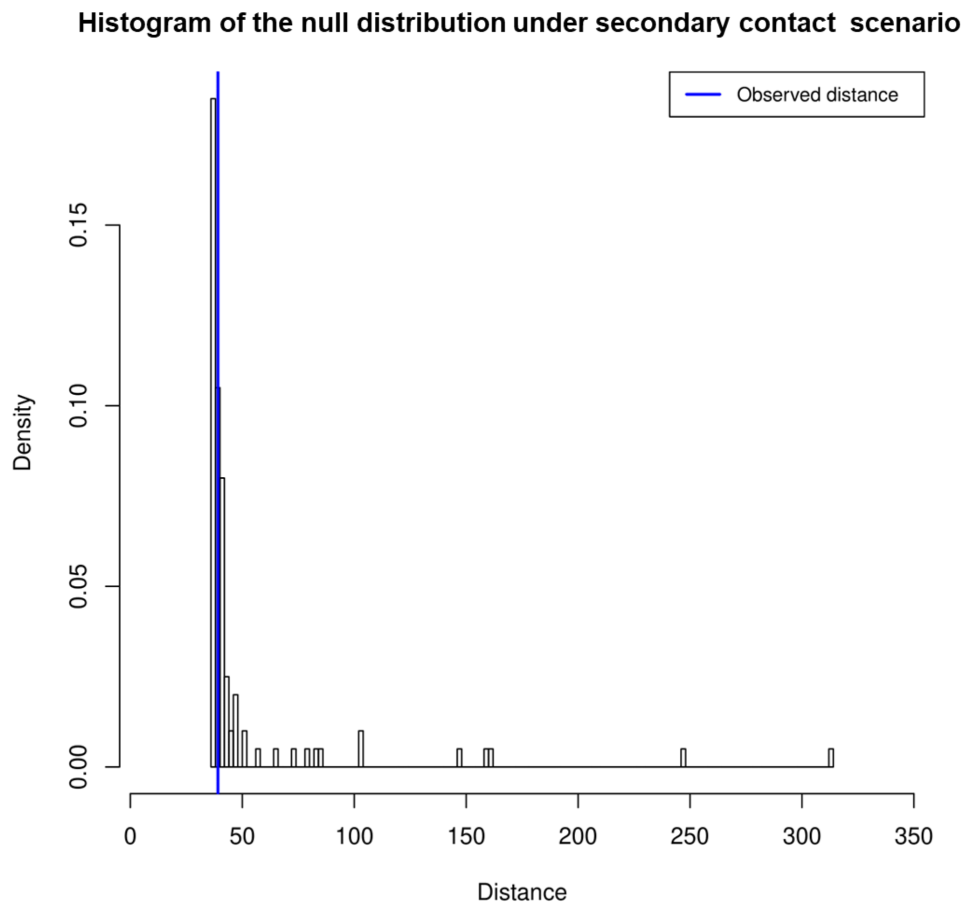

Figure S1. Histogram of the distribution under secondary contact (SC) scenario and the observed value.

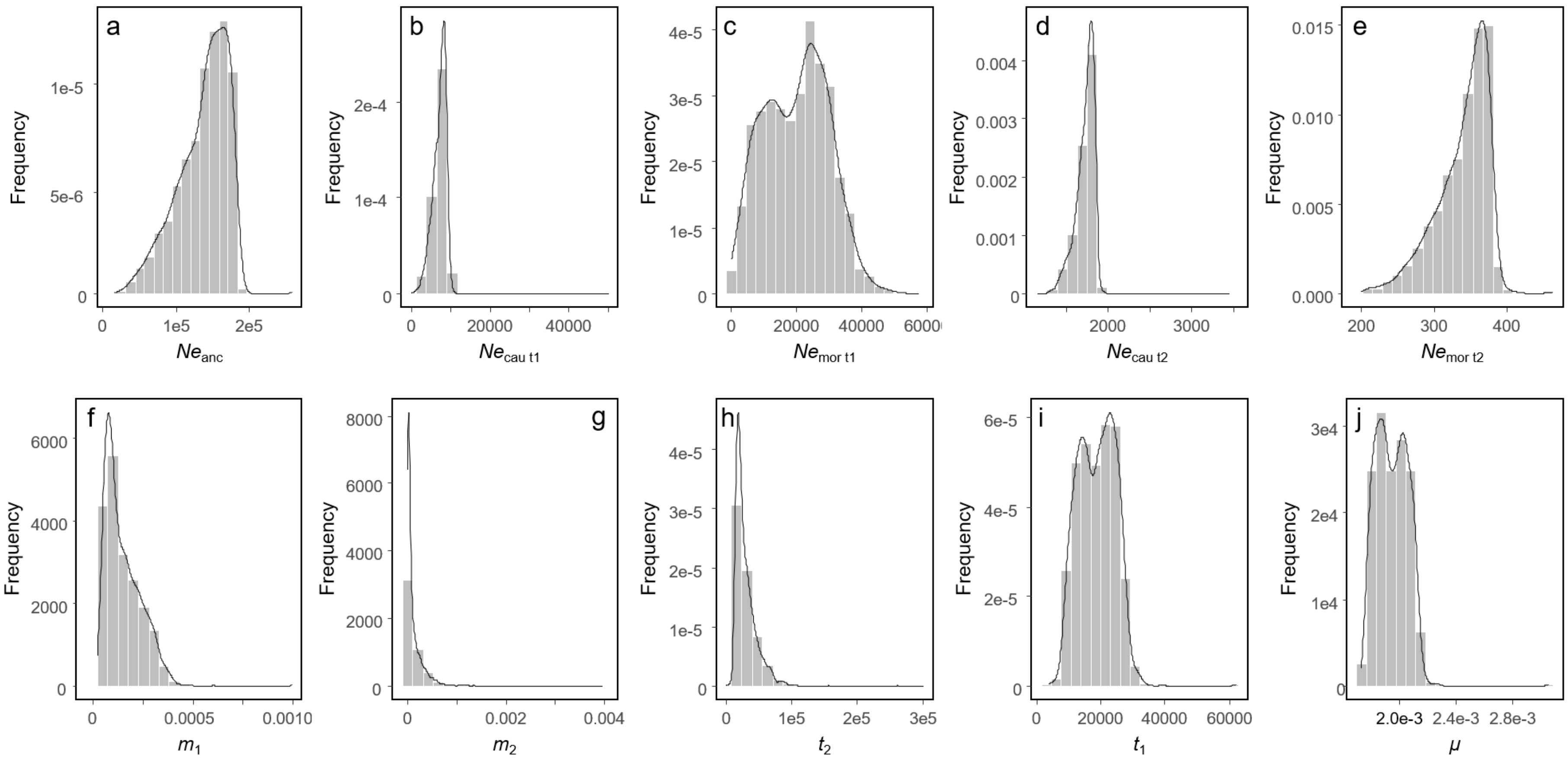

Figure S2. The frequency of marginal density of the best-fit scenario "secondary contact" (SC). **(a~e)** Effective population sizes of two maple species at t_1 ($Ne_{\text{cau t1}}$ and $Ne_{\text{mor t1}}$) and t_2 ($Ne_{\text{cau t2}}$ and $Ne_{\text{mor t2}}$) and their common ancestor (Ne_{anc}); **(f)** and **(g)** migration rates from *A. caudatifolium* to *A. morrisonense* (m_1) and the opposite direction (m_2); **(h)** and **(i)** divergence time between species (t_1) and the onset time of interspecific gene flow (t_2); **(j)** mutation rate.

Table S1. Sampling sites and sample size

Population	Lat.	Lon.	*A. caudatifolium*		*A. morrisonense*	
			**Alt. (m)**	***N***	**Alt. (m)**	***N***
RF	25°04'N	121°47'E	381-484	9	-	-
YMS	25°10'N	121°31'E	762-1021	33	-	-
LLS	24°41'N	121°24'E	1166-1317	18	1308-1577	19
JS	24°40'N	121°16'E	1197-1468	21	-	-
SKR	24°33'N	121°08'E	1546-1975	30	1933-2021	18
DXS	24°14'N	120°58'E	1680-2026	26	1855-2034	30
MF	24°05'N	121°10'E	2087-2283	31	2087-2321	31
DD	23°46'N	121°10'E	2196-2396	5	2236-2455	6
RL	23°42'N	120°55'E	1362-1642	17	-	-
TTC	23°31'N	120°54'E	1604-2388	31	1648-2350	31
ALS	23°28'N	120°51'E	1930-2404	22	2299-2540	30
STC	23°16'N	120°55'E	2324	1	2324	10
TJ	23°03'N	120°44'E	1467-1490	2	-	-
JBS	23°33'N	120°45'E	1306-2040	39	1280-1306	2
JSY	22°24'N	120°45'E	1257	18	-	-
MC	24°37'N	121°29'E	1106-1218	25	-	-
TPS	24°31'N	121°31'E	1566-1764	15	1471-1824	26
SY	24°20'N	121°19'E	1848-2000	31	1796-2039	25
TRK	24°11'N	121°20'E	2336-2987	27	2336-2700	32
LD	23°14'N	120°59'E	2033-2309	29	1939-2309	36

Table S2. Search ranges of parameters in ABC model

Parameter	Type	Distribution	Range
$Ne_{\mathrm{cau\ t2}}$	interger	unif	$100\text{-}10^4$
$Ne_{\mathrm{mor\ t2}}$	interger	unif	$100\text{-}10^4$
$\mathrm{N_{cau}}$	decimal	unif	0.01-100
$\mathrm{N_{mor}}$	decimal	unif	0.01-100
$\mathrm{N_{anc}}$	decimal	unif	0.001-1000
m_1	decimal	logunif	$10^{-9}\text{-}0.1$
m_2	decimal	logunif	$10^{-9}\text{-}0.1$
t_2	interger	logunif	$100\text{-}10^6$
t_1	interger	logunif	$100\text{-}10^6$
μ	decimal	logunif	$10^{-5}\text{-}0.001$
**Rule**			
$t_1 > t_2$			

$Ne_{\mathrm{anc}} = Ne_{\mathrm{mor\ t2}} \times \mathrm{N_{anc}}$; $Ne_{\mathrm{cau\ t1}} = Ne_{\mathrm{cau\ t2}} \times \mathrm{N_{cau}}$; $Ne_{\mathrm{mor\ t1}} = Ne_{\mathrm{mor\ t2}} \times \mathrm{N_{mor}}$

Table S3. Genetic diversity of each sampled population and species revealed by private allele frequencies, number of alleles per locus (*Na*), effective number of alleles per locus (N_e), the information index (*I*), the observed (*Ho*), expected (*He*) and unbiased expected heterozygosity (*uHe*), and Fixation index (*F*).

Population	*N*	Private allele freq.	*Na*		*Ne*		*I*		*Ho*		*He*		*uHe*		*F*	
			Mean	SE	Mean	SE	Mean	SE	Mean	SE	Mean	SE	Mean	SE	Mean	SE
A. caudatifolium																
YMS	29	0.0172	2.647	0.373	1.599	0.217	0.446	0.126	0.195	0.059	0.235	0.067	0.239	0.069	0.200	0.084
MF	22	0	1.941	0.315	1.359	0.146	0.300	0.098	0.122	0.045	0.172	0.056	0.177	0.057	0.268	0.072
TRK	24	0	2.353	0.308	1.453	0.141	0.415	0.094	0.180	0.049	0.232	0.053	0.237	0.055	0.293	0.104
SY	25	0.0208	2.235	0.379	1.425	0.186	0.352	0.107	0.148	0.049	0.189	0.056	0.193	0.057	0.285	0.099
TPS	11	0	2.176	0.404	1.507	0.202	0.383	0.119	0.182	0.061	0.213	0.064	0.223	0.067	0.149	0.093
MC	24	0.0208	2.529	0.493	1.471	0.220	0.361	0.125	0.146	0.051	0.180	0.063	0.184	0.064	0.111	0.046
JS	21	0	2.882	0.492	1.769	0.282	0.551	0.134	0.179	0.056	0.290	0.065	0.297	0.067	0.331	0.123
DXS	19	0.1316	4.059	0.949	2.905	0.751	0.764	0.214	0.172	0.052	0.342	0.087	0.352	0.090	0.288	0.110
RL	13	0.0385	3.765	0.860	2.754	0.604	0.760	0.208	0.209	0.055	0.356	0.087	0.371	0.091	0.218	0.117
TTC	26	0	2.588	0.403	1.386	0.147	0.381	0.098	0.183	0.044	0.200	0.050	0.204	0.051	0.074	0.082
ALS	19	0.1053	4.235	0.961	2.899	0.683	0.825	0.209	0.236	0.063	0.381	0.084	0.391	0.086	0.196	0.125
TJ	2	0	1.647	0.226	1.584	0.211	0.359	0.116	0.206	0.096	0.228	0.070	0.304	0.094	0.162	0.192
LD	26	0.0962	4.588	1.115	2.703	0.589	0.786	0.214	0.170	0.042	0.354	0.086	0.361	0.088	0.292	0.095
LLS	18	0.0833	3.882	0.790	2.373	0.473	0.729	0.191	0.203	0.057	0.338	0.082	0.348	0.085	0.277	0.095
RF	9	0	2.412	0.446	1.810	0.310	0.509	0.145	0.168	0.052	0.271	0.071	0.288	0.075	0.238	0.113
STC	1	0	1.176	0.095	1.176	0.095	0.122	0.066	0.176	0.095	0.088	0.048	0.176	0.095	-1.000	0.000
JSY	13	0.2307	2.882	0.685	2.172	0.482	0.556	0.183	0.080	0.035	0.273	0.083	0.284	0.086	0.618	0.098
SKR	28	0.0548	4.294	0.931	2.552	0.469	0.829	0.192	0.213	0.063	0.397	0.084	0.404	0.085	0.361	0.120
DD	5	0	2.235	0.349	1.934	0.309	0.534	0.146	0.105	0.051	0.297	0.079	0.335	0.089	0.612	0.108
JBS	36	0.0166	4.176	1.015	2.468	0.682	0.657	0.199	0.111	0.033	0.296	0.080	0.300	0.081	0.481	0.098
Total	371	-	8.588	1.857	2.024	0.309	0.799	0.180	0.169	0.044	0.341	0.073	0.341	0.073	0.366	0.082
A. morrisonense																
MF	28	0.0276	2.118	0.331	1.394	0.142	0.338	0.096	0.193	0.060	0.196	0.056	0.200	0.057	0.142	0.101
TRK	32	0.0185	3.059	0.449	1.601	0.204	0.487	0.121	0.177	0.057	0.254	0.063	0.258	0.064	0.380	0.101
SY	24	0.0416	2.471	0.298	1.483	0.168	0.402	0.107	0.154	0.050	0.219	0.061	0.224	0.062	0.234	0.077
TPS	26	0.0384	2.471	0.394	1.423	0.173	0.354	0.109	0.124	0.043	0.185	0.060	0.188	0.061	0.302	0.081
DXS	28	0.1071	2.235	0.315	1.466	0.141	0.388	0.098	0.170	0.056	0.228	0.060	0.232	0.061	0.295	0.103
TTC	30	0.0166	2.353	0.296	1.471	0.147	0.395	0.097	0.224	0.068	0.229	0.059	0.233	0.060	0.180	0.111
ALS	29	0	2.412	0.403	1.536	0.168	0.431	0.110	0.174	0.064	0.244	0.063	0.248	0.064	0.297	0.122
LD	36	0	3.647	0.658	1.584	0.153	0.551	0.117	0.191	0.051	0.282	0.059	0.285	0.060	0.370	0.086
LLS	17	0.0357	3.000	0.470	1.615	0.174	0.536	0.118	0.166	0.052	0.281	0.060	0.289	0.062	0.429	0.124
STC	10	0	1.824	0.300	1.407	0.149	0.317	0.106	0.241	0.083	0.186	0.062	0.196	0.065	-0.256	0.044
SKR	18	0	2.118	0.225	1.369	0.105	0.354	0.081	0.186	0.055	0.208	0.051	0.214	0.052	0.224	0.119
DD	6	0	1.882	0.241	1.461	0.127	0.386	0.098	0.196	0.069	0.234	0.059	0.256	0.065	0.267	0.131
JBS	2	0	1.294	0.143	1.204	0.108	0.160	0.077	0.147	0.071	0.103	0.048	0.137	0.064	-0.400	0.032
Total	286	-	6.235	1.130	1.578	0.175	0.525	0.115	0.180	0.050	0.261	0.062	0.261	0.062	0.398	0.088

Table S4. Pairwise F_{ST} between populations of two maple species. Bolds denotes the inter-population pairwise F_{ST} between species

		A. caudatifolium																				*A. morrisonense*											
		**YMS**	**MF**	**TRK**	**SY**	**TPS**	**MC**	**JS**	**DXS**	**RL**	**TTC**	**ALS**	**TJ**	**LD**	**LLS**	**RF**	**STC**	**JSY**	**SKR**	**DD**	**JBS**	**MF**	**TRK**	**SY**	**TPS**	**DXS**	**TTC**	**ALS**	**LD**	**LLS**	**STC**	**SKR**	**DD**
A. caudatifolium	**MF**	0.051																															
	**TRK**	0.058	0.039																														
	**SY**	0.053	0.049	0.036																													
	**TPS**	0.045	0.070	0.058	0.036																												
	**MC**	0.038	0.055	0.054	0.044	0.029																											
	**JS**	0.056	0.074	0.062	0.052	0.047	0.049																										
	**DXS**	0.124	0.133	0.113	0.120	0.124	0.108	0.075																									
	**RL**	0.150	0.142	0.132	0.136	0.175	0.152	0.104	0.056																								
	**TTC**	0.066	0.042	0.036	0.060	0.065	0.054	0.061	0.110	0.103																							
	**ALS**	0.142	0.136	0.124	0.129	0.141	0.147	0.083	0.050	0.028	0.095																						
	**TJ**	0.236	0.242	0.203	0.214	0.268	0.255	0.181	0.123	0.131	0.163	0.099																					
	**LD**	0.092	0.091	0.075	0.081	0.086	0.087	0.054	0.030	0.040	0.065	0.027	0.084																				
	**LLS**	0.072	0.089	0.078	0.079	0.069	0.059	0.044	0.033	0.070	0.079	0.064	0.129	0.032																			
	**RF**	0.095	0.123	0.105	0.097	0.117	0.112	0.080	0.066	0.108	0.115	0.092	0.215	0.058	0.056																		
	**STC**	0.303	0.329	0.300	0.260	0.373	0.336	0.273	0.215	0.224	0.257	0.228	0.421	0.186	0.223	0.306																	
	**JSY**	0.149	0.148	0.153	0.131	0.173	0.175	0.128	0.115	0.102	0.127	0.067	0.161	0.064	0.109	0.136	0.306																
	**SKR**	0.086	0.095	0.080	0.076	0.068	0.083	0.041	0.050	0.076	0.088	0.052	0.123	0.033	0.038	0.057	0.222	0.077															
	**DD**	0.137	0.126	0.131	0.134	0.168	0.158	0.114	0.069	0.099	0.130	0.076	0.182	0.051	0.070	0.086	0.283	0.098	0.062														
	**JBS**	0.122	0.112	0.125	0.118	0.120	0.102	0.099	0.064	0.082	0.103	0.071	0.166	0.044	0.057	0.090	0.220	0.075	0.063	0.073													
A. morrisonense	**MF**	**0.356**	**0.358**	**0.345**	**0.386**	**0.364**	**0.370**	**0.371**	**0.368**	**0.362**	**0.313**	**0.360**	**0.463**	**0.322**	**0.302**	**0.420**	**0.570**	**0.440**	**0.315**	**0.420**	**0.375**												
	**TRK**	**0.292**	**0.294**	**0.280**	**0.314**	**0.299**	**0.303**	**0.308**	**0.307**	**0.298**	**0.257**	**0.300**	**0.371**	**0.265**	**0.249**	**0.352**	**0.451**	**0.342**	**0.263**	**0.330**	**0.309**	0.035											
	**SY**	**0.335**	**0.337**	**0.319**	**0.357**	**0.343**	**0.349**	**0.341**	**0.336**	**0.324**	**0.313**	**0.324**	**0.420**	**0.314**	**0.298**	**0.383**	**0.519**	**0.372**	**0.293**	**0.358**	**0.347**	0.024	0.031										
	**TPS**	**0.349**	**0.358**	**0.345**	**0.376**	**0.359**	**0.366**	**0.363**	**0.353**	**0.342**	**0.334**	**0.344**	**0.448**	**0.330**	**0.310**	**0.397**	**0.535**	**0.416**	**0.307**	**0.400**	**0.357**	0.079	0.039	0.061									
	**DXS**	**0.364**	**0.368**	**0.344**	**0.388**	**0.372**	**0.380**	**0.368**	**0.362**	**0.363**	**0.324**	**0.359**	**0.460**	**0.324**	**0.301**	**0.411**	**0.569**	**0.408**	**0.309**	**0.390**	**0.381**	0.035	0.041	0.023	0.085								
	**TTC**	**0.333**	**0.343**	**0.319**	**0.346**	**0.336**	**0.347**	**0.333**	**0.331**	**0.323**	**0.332**	**0.320**	**0.419**	**0.314**	**0.300**	**0.381**	**0.521**	**0.378**	**0.306**	**0.367**	**0.348**	0.062	0.037	0.034	0.062	0.044							
	**ALS**	**0.384**	**0.397**	**0.371**	**0.406**	**0.393**	**0.405**	**0.381**	**0.372**	**0.364**	**0.342**	**0.359**	**0.477**	**0.330**	**0.315**	**0.423**	**0.555**	**0.420**	**0.322**	**0.405**	**0.388**	0.068	0.048	0.038	0.072	0.053	0.015						
	**LD**	**0.352**	**0.364**	**0.340**	**0.375**	**0.361**	**0.370**	**0.344**	**0.329**	**0.327**	**0.315**	**0.319**	**0.430**	**0.294**	**0.281**	**0.378**	**0.519**	**0.378**	**0.285**	**0.360**	**0.342**	0.068	0.040	0.044	0.058	0.053	0.024	0.015					
	**LLS**	**0.329**	**0.339**	**0.312**	**0.350**	**0.338**	**0.345**	**0.325**	**0.299**	**0.302**	**0.338**	**0.296**	**0.381**	**0.290**	**0.275**	**0.333**	**0.510**	**0.340**	**0.272**	**0.321**	**0.316**	0.073	0.056	0.057	0.070	0.062	0.068	0.053	0.035				
	**STC**	**0.438**	**0.463**	**0.404**	**0.476**	**0.457**	**0.469**	**0.403**	**0.401**	**0.397**	**0.371**	**0.393**	**0.519**	**0.358**	**0.336**	**0.452**	**0.642**	**0.451**	**0.347**	**0.437**	**0.415**	0.063	0.045	0.040	0.063	0.061	0.022	0.029	0.043	0.079			
	**SKR**	**0.379**	**0.398**	**0.358**	**0.400**	**0.389**	**0.403**	**0.368**	**0.365**	**0.368**	**0.325**	**0.364**	**0.467**	**0.328**	**0.307**	**0.403**	**0.600**	**0.420**	**0.306**	**0.406**	**0.395**	0.070	0.045	0.053	0.065	0.052	0.046	0.052	0.042	0.038	0.056		
	**DD**	**0.369**	**0.375**	**0.359**	**0.400**	**0.381**	**0.387**	**0.370**	**0.359**	**0.351**	**0.324**	**0.346**	**0.460**	**0.316**	**0.299**	**0.414**	**0.557**	**0.429**	**0.310**	**0.411**	**0.364**	0.059	0.053	0.035	0.091	0.055	0.046	0.040	0.034	0.057	0.077	0.071	
	**JBS**	**0.505**	**0.531**	**0.466**	**0.557**	**0.532**	**0.538**	**0.469**	**0.463**	**0.460**	**0.423**	**0.456**	**0.594**	**0.415**	**0.386**	**0.518**	**0.718**	**0.556**	**0.399**	**0.532**	**0.470**	0.160	0.111	0.084	0.165	0.136	0.093	0.109	0.094	0.130	0.175	0.146	0.100

Table S5. Model comparison between four scenarios with the Bayes factor

	**CM**	**AM**	**SC**	**SI**
CM	1	2.4399	0.7379	72.7905
AM	0.4098	1	0.3024	29.8330
SC	1.3552	3.3066	1	98.6451
SI	0.0137	0.0335	0.0101	1

Table S6. Estimates of model parameters for the four scenarios through approximate Bayesian computation

	Ne_{anc}	$Ne_{cau\ t1}$	$Ne_{mor\ t1}$	$Ne_{cau\ t2}$	$Ne_{mor\ t2}$	m_1	m_2	t_1	t_2	μ
SC										
mode	149670	8232	24220	1792	366	8.0E-5	2.0E-5	22925	18870	1.9E-4
mean	125338	6938	18696	1722	339	1.5E-4	1.2E-4	18526	28881	1.9E-4
median	135659	7421	20155	1749	348	1.3E-4	5.0E-5	18687	23748	1.9E-4
Wt. 2.5% perc.	39472	2848	2495	1441	256	5.0E-5	0	9349	14067	1.8E-4
Wt. 97.5% perc.	187873	9750	38419	1854	381	3.3E-4	5.7E-4	27664	66799	2.1E-4
CM										
mode	1931504	73878	255311	3886	3896	4E-08	6.2E-6	6356	425	3.4E-4
mean	1424233	52035	226709	3149	3607	1.4E-07	7.5E-6	5451	663	5.4E-4
median	1552088	57047	237125	3292	3698	9E-08	7.4E-6	5497	556	4.8E-4
Wt. 2.5% perc.	382271	9568	143124	1065	2706	1E-08	5.2E-6	3033	320	2.3E-4
Wt. 97.5% perc.	2281474	99174	277702	4844	4084	5.3E-07	1.04E-5	7603	1540	1.08E-03
AM										
mode	125863	310881	2529	6130	5944	2.2E-06	0.005	3502	555	2.9E-4
mean	89787	197772	2080	4542	5633	0.001	0.027	16006	12805	9.0E-4
median	95656	217563	2219	4759	5757	1.5E-5	0.012	10597	1280	6.6E-4
Wt. 2.5% perc.	8808	7812	850	1051	4513	0	0.001	380	117	1.4E-4
Wt. 97.5% perc.	177600	522977	2939	7106	6163	0.013	0.119	58411	111091	2.91E-03
SI										
mode	2145651	293434	168879	8814	2541	-	-	18179	1324	4.0E-5
mean	1298101	250791	121772	5144	2485	-	-	181421	18188	2.2E-4
median	1290808	253427	123777	5210	2485	-	-	61372	1571	1.1E-4
Wt. 2.5% perc.	4502	943	612	357	192	-	-	516	125	1.0E-5
Wt. 97.5% perc.	4768009	939528	458900	9738	4737	-	-	884502	168403	8.9E-4
